# Associations between children’s physical literacy and well-being: is physical activity a mediator?

**DOI:** 10.1186/s12889-022-13517-x

**Published:** 2022-06-29

**Authors:** Paulina S. Melby, Glen Nielsen, Jan Christian Brønd, Mark S. Tremblay, Peter Bentsen, Peter Elsborg

**Affiliations:** 1 Department of Nutrition, Exercise and Sports, University of Copenhagen, Copenhagen N, Denmark; 2 Health Promotion, Steno Diabetes Center Copenhagen, the Capital Region of Denmark, Herlev, Denmark; 3 Department of Sports Science and Clinical Biomechanics, Research Unit for Exercise Epidemiology, Centre of Research in Childhood Health, University of Southern Denmark, Odense, Denmark; 4 Children’s Hospital of Eastern Ontario Research Institute, Ottawa, Canada; 5 Department of Pediatrics, University of Ottawa, Ottawa, Canada; 6 Center for Clinical Research and Prevention, Copenhagen University Hospital – Bispebjerg and Frederiksberg, Frederiksberg, Denmark; 7 Department of Geosciences and Natural Resource Management, University of Copenhagen, Frederiksberg C, Denmark

**Keywords:** CAPL-2, Childhood, Mental health, SEM

## Abstract

**Background:**

Physical literacy (PL) is a multi-dimensional concept that provides a holistic understanding of movement and physical activity. PL contains an affective, a physical, and a cognitive domain, which together lay the foundation for the individual’s capacity and the tendency for participating in physical activities currently and throughout life. PL is increasingly regarded as a ‘cause of the causes’ to health promotion. Cross-sectional studies have shown associations between children’s PL, physical activity behaviours, and well-being. This study aims to examine the associations between Danish children’s PL and their physical and psychosocial well-being and whether the associations are mediated by moderate- to vigorous intensity physical activity (MVPA).

**Methods:**

Cross-sectional data from Danish schoolchildren aged 7–13 years were collected in Jan-Dec 2020 in the Danish Assessment of Physical Literacy (DAPL) project. PL was assessed with the DAPL which measures the affective, cognitive, and physical domains of PL. MVPA (min/day) was measured with accelerometers (Axivity), psychosocial well-being was measured with The Strengths and Difficulties Questionnaire, and physical well-being was measured with the KIDSCREEN questionnaire. Structural equation models were constructed with PL and MVPA as predictors of physical well-being and four aspects of psychosocial well-being.

**Results:**

A positive moderate association between PL and physical well-being, partly mediated by MVPA was observed. PL was positively associated with the positive aspects of psychosocial well-being and negatively associated with the negative aspects (behaviour problems). None of the associations between PL and aspects of psychosocial well-being were mediated by MVPA.

**Conclusions:**

The study contributes to evidence on the link between PL, physical activity, and health outcomes. The study found beneficial relations between PL and physical and psychosocial well-being. MVPA mediated part of the relationship between PL and physical well-being but not psychosocial well-being.

**Supplementary Information:**

The online version contains supplementary material available at 10.1186/s12889-022-13517-x.

## Background

Populations’ engagement in healthy physical activity (PA) through life is important for limiting the burden of non-communicable diseases [1]. Literature reviews also show that PA in general benefits different aspects of children’s well-being [2–4]. However, many children and youth do not meet the recommendations and guidelines for health-enhancing PA; neither in Denmark [5] nor globally [6]. This may be partly due to the challenge that extrinsic motives for participating in PA, such as improved health, have shown to be insufficient for maintaining healthy movement behaviour [7, 8] and that policy and programs aimed at increasing PA (e.g. the school setting) often result in small or non-significant effects that do not last [9, 10]. The psychosocial well-being of the general Danish population has decreased across all genders and age groups during recent years [11]. Poor psychosocial well-being is particularly evident among youth were 34% of females, and 21% of males report low psychosocial well-being [11].

**Fig. 1:**
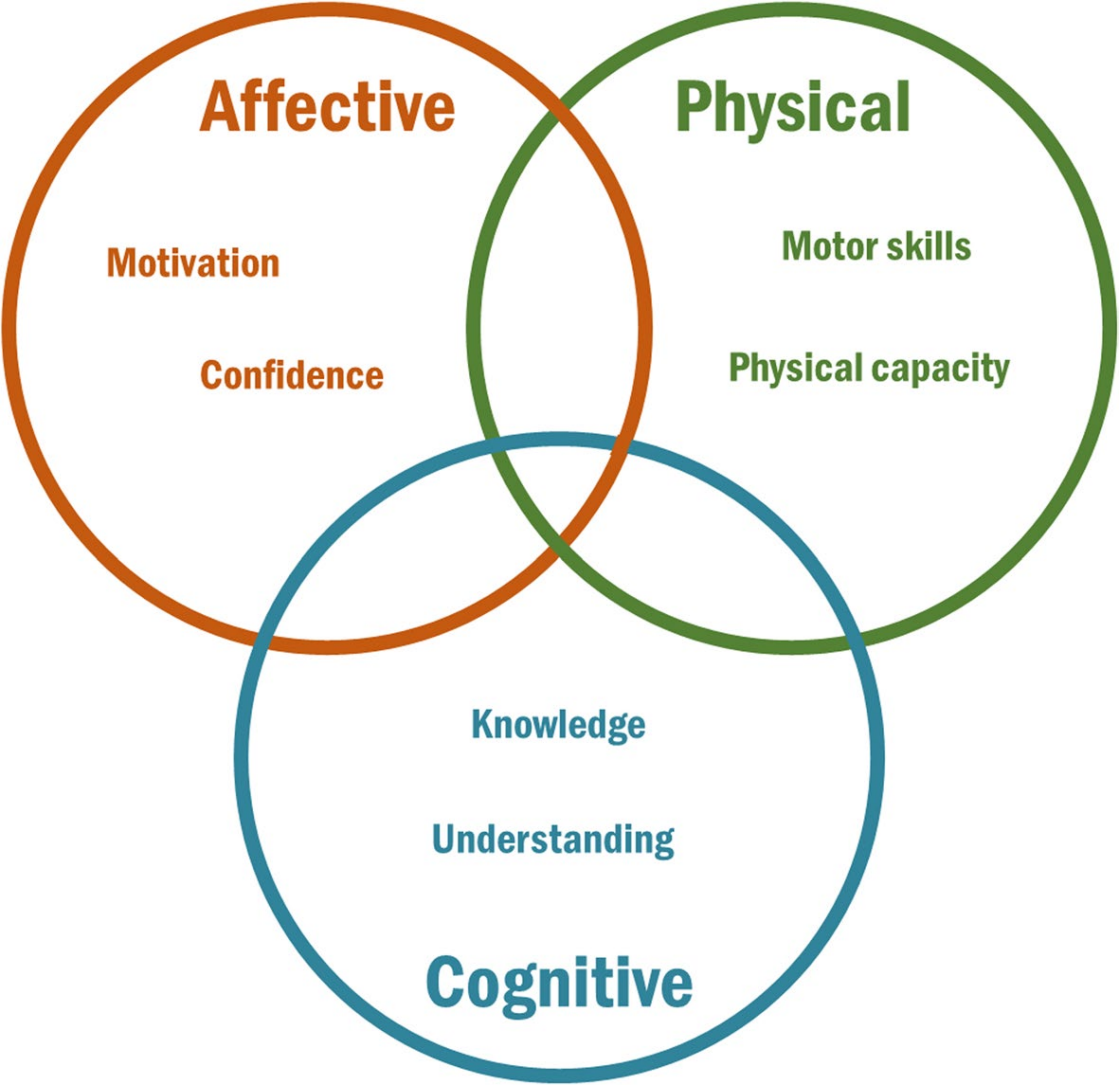
Ven diagram of physical literacy. Figure 1 shows the three domains of physical literacy and the elements of each domain [15]. The cognitive, affective and physical resources and attributes will in synergy enable physical activities through life

The potential of physical literacy (PL) for PA- and health promotion (e.g. psychosocial well-being) has gained attention recently [12]. PL is a comprehensive, multi-component concept describing important individual prerequisites for participating in and adhering to PA throughout the life course [13]. Different definitions of PL exist. One defines PL as consisting of personal cognitive, affective, and physical components that enable participation in PA throughout life [14]. Thus, PL is the personal abilities and dispositions that enable individuals to be physical active in different contexts (please, see Fig. 1 for a model of PL domains). Cross-sectional studies have shown correlations between children’s PL and PA behaviours [16], sedentary behaviour [17], cardiorespiratory fitness [18], screen time [17], resilience [19], active school transport [20], and weight status [21]. Studies have also shown an association between PL and indicators of children’s general health [21, 22]. Collectively, these findings suggest PL is an important potential health promotor to study.

Well-being is one important aspect of health and positive child development [23, 24]. Like PL, well-being is often defined as multidimensional, relying on and reflecting physical, affective, and social processes [25, 26]. However, to our knowledge, only one study has investigated the associations between PL and aspects of children’s well-being [22]. The authors observed, that higher PL in children was associated with favourable health indicators such as body fat, fitness, and quality of life, with the relationships between PL and aerobic fitness being mediated by daily moderate- to vigorous intensity PA (MVPA) [22]. However, there remains a paucity of research on how PL is associated with psychosocial health and well-being and further evidence is needed to advance our understanding on the associations among PL, PA, and health.

According to the self-determination theory (SDT) and SDT based research, motivation and wellbeing are closely related [27]. Since the affective domain of PL includes autonomous motivation, which is positively related to wellbeing and because PL is thought to enable PA, it is likely that PL could be a determinant of wellbeing. Further, according to the theory of PL, children with high PL increases their possibility of having positive experiences in PAs [13], and thus their possibility to feel well-being in PA contexts. It has been shown that the effects of motivation and psychosocial well-being can transfer across related contexts [28] and from the contextual to a more global level [29]. In sum, children with high PL will be more likely to feel well during PA’s which may affect their general well-being.

**Fig. 2:**
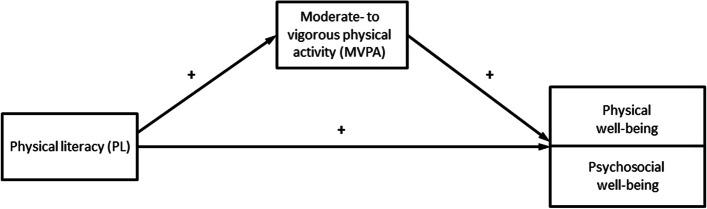
Hypothesized paths between study variables. Figure 2 shows the theorized models, with physical well-being and aspects of psychosocial well-being as the outcomes

Therefore, the objectives of this study are to a) investigate the association between PL and different aspects of psychosocial and physical well-being and b) investigate to what extent the associations are mediated by level of MVPA. We hypothesized that children’s PL would be associated with their daily level of PA and well-being and that the relation between PL and well-being would be partly mediated by daily PA (the hypothesized paths are shown in Fig. 2).

## Methods

### Study design

This is a cross-sectional study, that uses participants from the Danish Assessment of Physical Literacy (DAPL) project. The complete design and methodology of the DAPL study are described elsewhere [30], and thus only variables used in the analysis of this paper are described in the following methods section. Briefly, the DAPL study aimed to translate and adapt the Canadian Assessment of Physical Literacy second edition, CAPL-2 [31, 32], into Danish language and context and assess the psychometric properties. Data collection was carried out from January to December 2020 at 12 schools in the Eastern part of Denmark (Sealand). The methodological quality of this study was evaluated by the STROBE statement (See Additional file 1).

The DAPL measurement protocol was carried out in line with the original CAPL-2 manual [33]. Physical tests and questionnaires were administered during two consecutive physical education classes, by trained research assistants. In the week in between the test days, pupils wore an accelerometer (for 8 days) to measure MVPA. On the evening of the day before the first physical education class and the evening of the last physical education class, text messages were sent to the parents with a link to two questionnaires measuring children’s well-being and parental socioeconomic status.

### Sampling and participants

The participants were recruited through cooperation with municipalities and schools across the Eastern part of Denmark (Sealand). Efforts were made to ensure representability by selecting and inviting 19 schools from school districts with different distributions of socioeconomic and ethnic backgrounds. The 19 schools were contacted directly or through their municipality. Fourteen schools agreed to participate but two withdrew later because of COVID-19. From the 12 participating schools, 52 classes from 1st to 6th grade participated in the study. Of the 1144 invited pupils, 948 provided parental consent (mean age 10.2 years) to participate in the study (83% consent rate) and 647 had data on one of the outcome variables in the SEM models and were included in the study. All pupils in all classes participated in the physical tests and questionnaires as they were part of the physical education curriculum on the given day.

### Measurements

#### Study concepts and definitions

In this study, PL was defined as consisting of the components; motivation and confidence, physical competencies, knowledge, and understanding. Most definitions of PL include these components. Some definitions also include a social dimension [34], which we consider to be integrated into each component. Other definitions include a behavioural component (often operationalized as daily PA) [35]. In this study, we consider and study PA as an outcome of PL. Additionally, one objective of this study was to investigate the mediation effect of PA. Therefore, the behavioural domain (pedometer step counts and self-reported activity) of the original CAPL-2 was not included in the PL score used for analysis in this study.

While, no universally accepted definition of well-being exists [26], we assign to a well-recognized multidimensional definition that includes psychological, physical, and social aspects [25]. This definition of well-being focuses on how well an individual is functioning in their daily life. By using this definition, well-being can be operationalized more meaningfully in child and youth community samples [26]. Thus, physical well-being is a general state of physical health and daily state of physical functioning, and psychological and social well-being is subjective experiences of pleasant and/or negative emotions about self and others. The term psychosocial well-being includes psychological and social well-being together.

#### Measurement of physical literacy

PL was assessed using the DAPL instrument [30] which stringently follows the original CAPL-2 manual [32]. Calculations of the scores for each test and questionnaire were done following the CAPL-2 manual, described in detail at the CAPL website [33]. The CAPL-2 has been translated and validated with success in other countries [31, 36, 37]. The validity and reliability of individual PL components were tested concurrently in the same sample [30].

For this analysis, the PL score was calculated as an aggregate of the three domains Physical Competence (maximum of 30 points), Motivation and Confidence (maximum of 30 points), and Knowledge and Understanding (maximum of 10 points), and ranges from 0 points to 70 points.

The physical competence domain score (maximum of 30 points) was calculated as an aggregate of the scores from the following three physical tests; a score for torso strength (0–10 points) assessed with The Plank Assessment of Torso Strength [38], a score for aerobic fitness assessed with The Progressive Aerobic Cardiovascular Endurance Run [39], and a score from The Canadian Agility and Movements Skill Assessment (0–10 points), which is a validated assessment battery of motor competence [40]. The Canadian Agility and Movements Skill Assessment measures the child’s motor proficiency in both manipulative and locomotor skills in movements that reflect movement skills a child ought to acquire at ages 8–12 years [41].

The motivation and confidence domain (maximum of 30 points) was assessed with a questionnaire and reflects the child’s confidence in their own ability to be physically active and their autonomous motivation for participation in PA. The components encapsulate scores from four subscales (with 4 items each) for adequacy, self-competence, predilection, and intrinsic motivation for PA and have shown good validity and reliability in children [42].

The knowledge and understanding domain (maximum of 10 points) was assessed with a questionnaire (10 items) and reflects the child’s knowledge about PA recommendations, aerobic fitness, and prerequisites required in specific sports genres [43].

#### Measurement of physical activity

Axivity AX3 accelerometers (Axivity Ltd., Newcastle upon Tyne, United Kingdom) were used to estimate the daily amount of MVPA. Sensitivity was set to +/− 8 g and sampling frequency to 50 Hz. The Axivity AX3 monitor has been validated in children in several studies (e.g. de Vries et al., 2006). The monitor is taped to the front of the left thigh [44] which allows the participant to wear the monitor while sleeping, bathing, and swimming providing a full 24-h recording. Participants were instructed to wear the monitors during 8 consecutive days, which has been recommended to estimate habitual PA [45] and to patch/replace the original tape to avoid it from falling off during the period. Data from the day of attachment and the day of removal were not included, which resulted in data from possible six days with 24 hours of recording.

The protocol for valid wear time and time spent in different intensities has been published elsewhere [46] and is thus only briefly described here. Time spent sitting, standing, walking, running, and biking have been validated in a similar population as in the present study and demonstrates excellent sensitivity and specificity above 85.8% [47]. Time spent lying is estimated by identifying the subject’s time to bed and out of bed using sitting and non-sitting behaviour during the time from 6 PM until 8 AM during Monday to Friday and from 6 PM until 11 AM during Saturdays and Sundays. Time to bed is identified as the last non-sitting activity bout from 6 PM until 2 AM which is longer than three minutes and followed by sitting event longer than 30 minutes. Time out of bed is identified as the first non-sitting period longer than 3 minutes identified after 4 AM. Non-wear time was marked as missing data by evaluating three signal features in combination (acceleration, temperature, and predefined awake time) [46]. Data were included in the final dataset if there was valid data for at least one weekend day and three weekdays with a maximum of two hours of non-wear. All outcome variables are averaged values that are harmonized using a 5/7 and 2/7 adjustment for week and weekend days, respectively. Time spent performing MVPA was estimated using accelerometer counts using 10-s epochs accounting for the elevated post-oxygen consumption during intermittent PA [48]. Cut-points for MVPA were established using a calibration study identifying and validating age-dependent intensity-specific counts thresholds. The thigh counts per minute cut-points for our age group were 4822 for moderate and 9143 for vigorous PA.

#### Measurement of physical well-being

Physical well-being was assessed with the subscale (5 items) from the KIDSCREEN parent version [49]. The subdimension physical well-being covers the level of the child’s PA (1 item), energy (1 item), and general health (2–3 items). An electronic parent version was distributed via text message along with the SDQ questionnaire. Parents were first asked; *In general, how would your child rate her/his health?* (excellent, very good, good, fairly, poor), and then they were asked to think about the last week and answer on a 5 point Likert scale (from poor/not at all/never to excellent/extremely/always); *Has your child felt fit and well?*; *Has your child been physically active (*e.g. *running, climbing, biking)*?; *Has your child been able to run well?*; and *Has your child felt full of energy?.*

#### Measurement of psychosocial well-being

Aspects of psychosocial well-being were assessed with the parent version of the Strengths and Difficulties Questionnaire (SDQ) [50], which measures behaviour conduct in everyday life, and thus is suitable for a healthy sample of children. A link to the SDQ parent version was distributed via text message to participants’ parents/legal guardians on the evening of the second testing day (day 8). The SDQ is a 25-item questionnaire developed for measuring mental health among children and adolescents and is widely used [51]. The SDQ has been thoroughly validated in large Danish samples [52, 53]. The questionnaire covers four negative aspects of psychosocial well-being included in the subscales: hyperactivity-inattention, conduct problems, emotional problems, and peer problems, and one positive aspect of psychosocial well-being included in the prosocial subscale [51]. All subscales are measured with five items answered with a five-point Likert scale. For analysis, we used an externalizing problems score aggregating the conduct problems scale and hyperactivity scales, and an internalizing problems score aggregating the emotional and peer problems scales. Further, we used the individual prosocial scale as this is not included in any of the combined scales. These subscales have been proposed to be appropriate in a homogenous community sample [54]. However, we decided to also use the total difficulties score aggregating the four negative scales which enable us to compare our results to other studies.

#### Measurement of socioeconomic status (SES)

Socioeconomic status (SES) was estimated with the Danish Occupational Social Class measurement, which assesses the highest social group level of the participant’s parents/guardians [55]. The Danish Occupational Social Class categorizes social class based on occupational skills and formal work qualifications based on the influence and power related to these. An electronic background questionnaire was distributed via text message to one of the participants’ parents/legal guardians on the evening of the day before the first testing day. The parent receiving the text message was asked to answer for themselves and the other parent. The social group variable (the highest of the two parents) was recoded into an SES variable ranging from class 1 (highest social class) to 6 (lowest social class), following the Danish Occupational Social Class protocol [55].

### Data analyses

Descriptive statistics, unpaired t-tests, bivariate correlations and, robust Cronbach’s alpha estimations were done in SPSS 25.0 [56]. McDonald’s Omega (ML) estimations were calculated in SPSS using Andrew Hayes’ macro [57]. Reliability was examined for the psychometric subscales, as well as for the three combined scales with values above 0.7 considered acceptable [58]. In the case where individual scales are used as outcome variables (i.e. physical well-being and pro-social scale), and since these are psychological latent variables measured by only five items, values above 0.6 were considered acceptable [59].

Associations between PL, MVPA, physical well-being, and the different aspects of psychosocial well-being (see hypothesized model in Fig. 2) were investigated through structural equation modelling (SEM) in Mplus [60]. We used maximum likelihood estimation with robust standard errors (MLR) to estimate missing values, which minimises selection bias. All models were adjusted for clustering effect by school classes. The distributions of study variables were inspected visually and were normally distributed. We followed recommended criteria for a good model fit: Tucker-Lewis index (TLI > 0.95), comparative fit index (CFI > 0.95), and root mean square error of approximation (RMSEA < 0.06) [61]. Adjustments were made for sex, age, and SES. Covariation between all exogenous variables was allowed. Significance tests were 2-tailed and *P*-values below 0.05 were considered statistically significant.

### Ethical considerations

Study procedures were assessed and approved by the regional ethics committee ‘*De Videnskabsetiske Komitéer, Region Hovedstaden*’ (journal number: 19088122). In Denmark, only biomedical research and research projects that entail a risk for participants can receive a Trial Registration Number through ethics review. Written information about the study was given to all school principals, teachers, and parents/guardians before the start of the study, and informed consent was obtained from the legal guardians of all participants. Study procedures were registered and approved by The Capital Region’s center for data reviews ‘*Videnscenter for Dataanmeldelser*’ (Reference: P-2019-659).

## Results

### Description of the sample and study variables

The mean age of the 948 study participants was 10.37 (SD = 1.52) years and 54% were girls. The number of participants with data, minimum, maximum, mean scores, and standard deviations for all variables, and subdomains of PL are reported in Table 1 (note that N for each variable varies). The SEM models only include participants with data for the outcome variable.

**Table 1:** Description of sample on PL, PA, and well-being variables

	N	Min.	Max.	Mean	SD	Skewness	Kurtosis	α	Ω
Knowledge & understanding (0–10)	574	0.00	10.00	6.74	2.08	−0.56	−0.09		
Motivation & confidence (0–30)	594	9.00	30.00	25.71	3.94	−1.09	0.80		
Physical competence (0–30)	524	4.50	30.00	18.95	5.85	−0.21	−0.60		
Physical literacy (0–70)	487	25.00	68.93	51.63	8.70	−0.27	− 0.14		
MVPA (min/day)	491	5.29	176.67	69.25	24.67	0.53	0.74		
Physical Well-being (0–5)	647	1.00	5.00	3.93	0.67	−0.66	0.96	.81	.82
Externalizing score (0–20)	647	0.00	15.00	3.95	3.30	0.88	0.21	.79	.82
Hyperactivity scale (0–10)	647	0.00	10.00	2.86	2.43	0.67	−0.37	.76	.81
Conduct scale (0–10)	647	0.00	8.00	1.09	1.35	1.72	3.89	.58	.56
Internalizing score (0–20)	647	0.00	16.00	3.36	3.10	1.26	1.77	.74	.75
Emotional scale (0–10)	647	0.00	10.00	2.31	2.14	1.02	0.80	.69	.70
Peer problems scale (0–10)	647	0.00	8.00	1.05	1.52	1.66	2.69	.62	.64
Total difficulties score (0–40)	647	0.00	30.00	7.31	5.42	1.17	1.61	.83	.83
Prosocial score (0–10)	647	4.00	10.00	8.87	1.27	−1.15	0.79	.52	.54

*Min.* Minimum, *Max.* Maximum, *SD* Standard deviation, ***α*** Cronbach’s alpha, Ω Omega (ML), *MVPA* Moderate- to vigorous physical activity

### Reliability

Reliability in terms of the internal consistency of the scales for well-being was examined with robust Cronbach’s alpha, and McDonald’s omega [62] as presented in Table 1. Reliability coefficients for the physical well-being scale were good, which has also been found in a similar sample [63].

Reliability coefficients for the peer problems scale, the prosocial scale, and the conduct scale were below acceptable values (Table 1), which is in consistency with coefficients in similar samples [64]. Item reduction did not improve the reliability for any of these scales. Since the total difficulties score and the externalizing and internalizing scales showed good reliability, they were chosen for use as outcomes for analysis. Acceptable values for individual 5-item scales were above 0.6, and thus, the reliability coefficient for the prosocial scale was close to acceptable.

### Study variables

To assess if missing data were associated with a systematic bias, we compared participants with missing data in the well-being measures due to parents failing to fill in the questionnaire (the largest source of dropout) with participants with complete data on well-being (Table 2). Participants with missing data were younger, come from lower social class, and had lower means in PL elements; Knowledge & Understanding, Physical Competence, as well as total PL score compared to the sample with complete data in the outcome variable. The two groups did not differ in Motivation & Confidence and MVPA.

**Table 2:** Comparison of participants with missing data on well-being and participants with complete data on age, physical literacy, and physical activity

	N		Mean (SD)	
	1	2	1	2
SES	163	576	2.93 (1.47) *	2.55 (1.40)
Age	302	646	9.92 (1.63) **	10.37 (1.52)
Knowledge & Understanding (0–10)	235	574	6.10 (2.25) **	6.74 (2.08)
Motivation & Confidence (0–30)	243	594	25.58 (4.35)	25.71 (3.94)
Physical competence (0–30)	195	524	16.09 (6.28) **	18.95 (5.85)
Physical literacy (0–70)	170	487	47.72 (9.00) **	51.63 (8.70)
MVPA (min/day)	156	491	71.13 (29.18)	69.25 (24.67)

1: Missing data in the outcome variable, due to parents/guardians not answering the questionnaire. 2: Complete data in outcome variables. SD: standard deviation; **p = 0, *p < 0.01. *SES* Socioeconomic status, *MVPA* Moderate- to vigorous physical activity

### Associations among study variables

Intercorrelations for the subdomains of PL, PA, and well-being are reported in Table 3. The total PL score correlates significantly with all outcome (well-being and PA) variables in the assumed favourable directions, whereas MVPA only correlates significantly with the physical well-being score, the internalizing score, and the total difficulties score.

**Table 3:** Variable inter-correlations (Pearson’s R)

	1	2	3	4	5	6	7	8	9	10
1. Age										
2. Knowledge & understanding	**.443**									
3. Motivation & confidence	**−.108**									
4. Physical competence	**.412**	**.351**	**.263**							
5. Physical literacy	**.328**	**.491**	**.646**	**.878**						
6. MVPA	**−.338**	−.058	**.305**	**.253**	**.293**					
7. Physical Well-being	**−.107**	−.015	**.327**	**.332**	**.376**	**.428**				
8. Externalizing score	−.049	**−.240**	−.07	**−.198**	**−.239**	.009	**−.122**			
9. Internalizing score	.026	**−.131**	**−.147**	**−.193**	**−.245**	**−.189**	**−.331**	**.431**		
10. Total difficulties score	−.015	**−.219**	**−.126**	**−.228**	**−.280**	−.103*	**−.264**	**.856**	**.835**	
11. Prosocial score	.017	.073	**.118**	.089*	**.124**	.016	**.136**	**−.307**	**−.155**	**−.276**

*SD* Standard deviation; bold text indicates *p*-value under 0.01, * indicates a *p*-value under 0.05, *MVPA* Moderate- to vigorous physical activity (min/day)

### Associations among PL, MVPA, and physical well-being

**Fig. 3:**
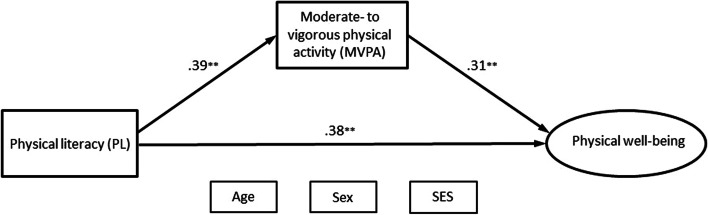
Path coefficients of the SEM with physical well-being as an outcome. Figure 3 shows path coefficients of the structural equation model with physical well-being as the outcome. All the parameters (β) were standardized and were statistically significant. Covariate variables are placed left of the model and without arrows to improve the visuality of the model. Covariation between all exogenous variables was allowed. See control paths in Table 4. SEM = structural equation model; MVPA = moderate- to vigorous physical activity; SES = socioeconomic status

Figure 3 shows the SEM with physical well-being as the outcome. All standardized path coefficients (β) and *p*-values are presented in Table 4.

**Table 4:** Information on path coefficients and R^2^ in the SEM with physical well-being as an outcome

**Paths**	**Std B**	**SE**	**P**
PL ➔ MVPA	0.39	0.05	.000
PL ➔ Physical well-being	0.38	0.06	.000
MVPA ➔ Physical well-being	0.31	0.05	.000
**Mediating paths**			
PL ➔ MVPA ➔ Physical well-being	0.12	0.03	.000
PL ➔ Physical well-being	0.38	0.06	.000
Total	0.50	0.05	.000
**Outcome Variable**	**R-square**	**SE**	**P**
Physical well-being	0.29	0.05	.000
MVPA	0.18	0.03	.000

Standardized regression weights (Std B), R squared (r^2^), standard error (SE), and *p*-values for paths in the structural equation model with physical well-being as the outcome. Controlled for age, sex, and SES. *PL* Physical literacy, *MVPA* Moderate- to vigorous physical activity

The SEM analysis showed that PL and MVPA were significantly and positively associated with physical well-being (Fig. 3). The model showed good model fit indices (CFI = 1.000, TLI = 1.000, RMSEA = .000). The path coefficients indicate that PL and MVPA are both of importance for children’s physical well-being. The association between PL and physical well-being was partly and significantly mediated by MVPA (β = 0.12, *p* = .000). There was a moderate direct association between PL and physical well-being (β = 0.38, *p* = .000). Overall, the model with PL, MVPA, and cofounding variables accounted for 29% of the variance in physical well-being.

### Associations between PL, MVPA, and aspects of psychosocial well-being

SEM was performed for each of the four psychosocial well-being outcome variables; Externalizing problems, Internalizing problems, Total difficulties score, and prosocial behavior. All standardized path coefficients (β) and *p*-values for each of the four models are presented in Table 5. All four models showed good model fit indices (For all models: CFI = 1.000, TLI = 1.000, RMSEA = .000).

**Table 5:** Information on path coefficients and R^2^ in the four SEM analyses with aspects of psychosocial well-being as outcomes

	Outcome: Externalizing			Outcome: Internalizing			Outcome: Total difficulties			Outcome: Prosocial		
**Paths**	**Std B**	**SE**	**P**	**Std B**	**SE**	**P**	**Std B**	**SE**	**P**	**Std B**	**SE**	**P**
PL ➔ MVPA	0.38	0.05	.000	0.38	0.05	.000	0.38	0.05	.000	0.38	0.05	.000
PL ➔ outcome	−0.31	0.05	.000	−0.24	0.07	.000	−0.32	0.06	.000	0.21	0.05	.000
MVPA ➔ outcome	0.16	0.05	.001	−0.07	0.05	.134	0.05	0.05	.308	−0.05	0.00	.000
**Mediating paths**												
PL ➔ PA ➔ outcome	0.06	0.02	.006	−0.03	0.02	.125	0.02	0.02	.329	−0.02	0.02	.352
PL ➔ outcome	−0.31	0.05	.000	−0.24	0.07	.000	−0.32	0.06	.000	0.21	0.07	.001
Total	−0.25	0.05	.000	−0.27	0.07	.000	−0.30	0.06	.000	0.19	0.05	.000
**R-square**	**R**^**2**^	**SE**	**P**	**R**^**2**^	**SE**	**P**	**R**^**2**^	**SE**	**P**	**R**^**2**^	**SE**	**P**
Outcome	0.12	0.03	.000	0.08	0.03	.011	0.10	0.03	.001	0.05	0.02	.030
MVPA	0.29	0.04	.000	0.29	0.04	.000	0.29	0.04	.000	0.30	0.04	.000
PL	0.18	0.03	.000	0.18	0.03	.000	0.18	0.03	.000	0.18	0.03	.000

Standardized regression weights (Std B), R squared (r^2^), standard error (SE), and p-values for paths in the structural equation model with aspects of psychosocial well-being as the outcomes. Controlled for age, sex, and socioeconomic status. *PL* Physical literacy, *MVPA* Moderate- to vigorous physical activity

The SEMs showed that PL was significantly and negatively associated with the three negative aspects of psychosocial well-being, and significantly and positively associated with the prosocial score, as we hypothesized (Fig. 2). There was a small detrimental association between MVPA the externalizing score (β = .16, *p* = .001) and a very small association between MVPA and the prosocial score (β = −.05, *p* = .000).

No mediation was found for any of the aspects of psychosocial well-being. We observed the strongest associations between PL and the total difficulties score (β = −.32, *p* = .000), and the externalizing score (β = −.31, *p* = .000), while we observed lower associations between PL and the internalizing score (β = −.24, *p* = .000) and the prosocial score (β = .21, *p* = .000). A significant mediating path was observed in the model with externalizing as the outcome variable, but the directions of the associations were opposite, and thus, were not considered.

Overall, PL, MVPA, and the cofounding variables accounted for 12% of the variance in the externalizing score (*p* = .000), 8% of the variance in the internalizing score (*p* = .011), 10% of the variance in the total difficulties score and 5% (*p* = .001) of the variance in the prosocial score (*p* = .000).

## Discussion

The results of this study indicate that PL is associated with important aspects of children’s well-being. First, we observed a positive moderate association between PL and physical well-being which was partly mediated by MVPA. The observed association between MVPA and physical well-being was positive and moderate indicating that PL is important for children’s physical well-being and that some of this relationship works through the level of PA. It is worth noticing that some of the items in the latent construct physical well-being are related to being active in general (e.g. “*Has your child been physically active?*” and “*Has your child felt full of energy?*”) and to cardiorespiratory fitness (i.e. “*Has your child been able to run well?*”). It is, therefore, perhaps not surprising that MVPA is associated with this construct of physical well-being. A high similar correlation between the KIDSCREEN physical well-being subdomain and objectively measured MVPA were observed in a similar sample [65].

Secondly, we observed a beneficial association between PL and all aspects of psychosocial well-being, with β-values ranging from .21 to −.32, and with no mediating role of MVPA. These results are in line with a study among 222 Canadian schoolchildren (mean age: 10.7 ± 1.0 years) where the authors observed a direct effect from PL to Health-Related Quality of Life (HRQoL), but no effect of MVPA on HRQoL, and no mediating effect of MVPA [22]. The latent construct HRQoL was measured with four subdomains of which three are comparable to the aspects of psychosocial well-being measured with the SDQ. The fourth subdomain corresponds to the physical well-being subdomain of KIDSCREEN.

We observed a detrimental association between MVPA and externalizing symptoms, which has been found in similar samples [66, 67]. This association may be explained by the nature of externalizing and especially hyperactive behaviour, resulting in higher levels of accelerometer measured daily total PA. Further, we observed a very weak detrimental association between MVPA and prosocial behaviour (β = −.05) and found no associations between MVPA and the remaining aspects of psychosocial well-being or the total difficulties score. Other studies have similarly found no association between MVPA and the SDQ measured total difficulties score [66–69]. Contrary to our results, these studies observed a beneficial association between MVPA and the internalizing subscale. A reason why we didn’t see this association in our study could be that we included PL in our models and/or that such relationships vary among population groups.

Together, the non-significant or detrimental associations between MVPA and different aspects of psychosocial well-being, and the beneficial association between PL and psychosocial well-being with or without controlling for MVPA in the model indicate that PL may have a positive impact on children’s general well-being. However, studies with an experimental design are needed to confirm this.

In general, it is difficult to compare results across studies investigating psychosocial well-being, as there is no universal definition to the term. This is perhaps a reason why systematic reviews and meta-analyses find mixed results of PA interventions on well-being [26, 70]. A review focusing on objectively measured levels of PA and the relation to different aspects of well-being in children and youth found some support for beneficial associations between MVPA and health indicators such as quality of life, pro-social behaviour, and psychological distress [71]. A large review of reviews examined the relationships between PA, depression, and anxiety in children and adolescents. The authors concluded that observational evidence indicates a beneficial association between PA and depression ranging from null to small, and very small to moderate effect sizes for PA on anxiety [2]. In this study, we only observed associations from PA to two aspects of psychosocial well-being. In our study, the construct psychosocial well-being is based on a definition and measure of psychological and social well-being as function in the everyday life measured by problem and well-functioning behaviour, and thus, it is problematic to compare it to constructs like depression and anxiety.

A reason why we observed an association between PL and psychosocial well-being, independent of PA could be that the affective elements of PL, motivation, and confidence, have an impact beyond PA contexts as described in Vallerands hierarchical principle of motivation [29]. As described in self-determination theory [72], well-being and intrinsic motivation are interlinked such that when the basic psychological needs (feeling of autonomy, confidence, and belonging) are satisfied the individual will experience well-being in an activity which is necessary for sustained intrinsic motivation for the activity [27]. According to the theory of PL [13], when an individual’s PL is positively developed in a movement context, all of the elements are positively stimulated including the affective elements motivation and confidence. Thus, children with greater PL score increase the possibility to have positive experiences in PAs [13], and thus increases the possibility to feel well-being in PA’s which is a part of their general well-being. It has been suggested that the effects of motivation and healthy functioning/well-being can transfer across related contexts [28]. Thus, children with greater PL will be more likely to feel well during PA’s and the effect may cross to other contexts of the child’s everyday life.

Even though the models only explained a small part of the variance in the outcomes for psychosocial well-being (5–12%), the results are relevant, as PL seems important for children’s psychosocial well-being beyond its association to MVPA and because both well-being and development of life competencies are the core purpose of schools in many countries.

### Implications for practice, policy, and research

First, findings from this study respond to the call for more research on the link between PL and physical, psychological, and social health, and provide more support for the assumption that PL is associated to important health outcomes in children. Second, the null finding of MVPA on aspects of psychosocial well-being along with the positive relation to PL supports the idea that helping children develop their PL is more beneficial for general long-term health than a narrow focus on increasing their levels of PA. This speaks to a need of shifting the focus from the amount and intensity of PA to quality and enjoyment when encouraging children to move. Health authorities and policy makers should consider including PL development in national PA guidelines and aims, so these contain guides on how to motivate and enable children to be active instead of the current sole focus on the amount of movement. According to this study and other PL research, it seems important that children are provided ample opportunities to engage in PAs that develop the motor-skills, fitness, understanding, confidence and autonomous motivation that enable them to be physically activity in different contexts. To achieve this, activities should be diverse to develop a broad spectrum of motor competencies, appropriately challenging to provide experiences of mastery/competence and hence confidence and motivation. It has also shown to be important for the development of well-being and autonomous motivation for PA that the social environments of the activities are focused on task solving/learning instead of results/competition as well as on giving social and autonomy support [73].

School leaders, educators, coaches and families all play a crucial role in promoting and developing PL in children. The focus should be on providing the best conditions for all children to acquire competences and attitudes that they can draw upon when engaging in movement and PA’s.

### Future research, strengths and limitations

There are some strengths and limitations connected to the design and methods of this study. The amount of missing data should be considered a limitation. The highest amount of missing data was in the outcome variables about well-being (68% response rate). This might be due to that these questionnaires were sent to the parents on the last day of the data collection period, however, the response rate for the questionnaire sent on the first day was only a little better (78%). From the dropout analysis (Table 2) it was evident that the participants with missing data in the outcome variables scored 7.5% lower in PL but did not differ in MVPA. Missing data in the PL elements were mostly due to children not attending school on one or both test days. Missing data in the PA measure were due to children removing the monitor after a few days, and to children who lost the monitor or forgot to return it. Nevertheless, for such direct measures used in this study the final sample size is rather large and should be considered a strength.

A strength of the study is the use of objective assessment of participants’ PA [74] with the use of skin-taped accelerometer mounting to reduce some of the limitations that are usually connected to this device [44]. The high number of participants (73%) with valid PA data supports the strength of this method. However, when using accelerometers alone, information about context, setting, and characteristics of the activities are absent, which could be important to understand the association between PA and psychosocial well-being.

The assessment of physical and psychosocial well-being also has some limitations. Physical well-being was assessed by the subdomain of the KIDSCREEN consisting of items that are also part of the construct PA, which perhaps makes it less suitable for exploring associations to MVPA.

The SDQ questionnaire used to measure psychosocial well-being has been criticized for being less suitable to measure the variance within a normal/healthy sample. This instrument was first developed to identify children with difficulties [50], which introduces the risk of a flooring effect, and thus loss of variance, when used in a community sample. Further, the reliability values for the individual prosocial scale were below acceptable values and thus, results related to this outcome should be considered with additional caution. Nevertheless, using the subscales for analysis brings additional information in the explored relationship among PL, PA, and different aspects of psychosocial well-being.

The use of the structural equation model in the investigation of the associations must also be considered a strength, as this method of analysis, minimises selection bias by imputing missing values by maximum likelihood estimation.

The main limitation of the study is perhaps the cross-sectional design, which introduces uncertainty about the direction or causality of the association. We must consider the possibility that children that thrive in general also thrive in the context of PA’s and thus have higher motivation, confidence, motor skills, etc. Future research studies with longitudinal and experimental designs are needed to inform us on the direction of the associations examined in this study.

## Conclusion

This study contributes to the scarce literature on associations between PL and health. The findings bring novel knowledge about the relation between children’s PL and physical and psychosocial well-being and the mediating role of PA. We hypothesized, that PL was associated with physical well-being and aspects of psychosocial well-being in children and found positive beneficial associations for all investigated associations. We further hypothesized, that PA had a mediating role in the associations, but found that this was only the case for the relationship between PL and physical well-being. Results from this study contribute to the existing evidence that PL is related to several health outcomes [14] by indicating that PL is important for children’s psychosocial well-being beyond its association to MVPA.

## Supplementary Information

**Additional file 1.**

## Data Availability

A dataset is available from the corresponding author upon reasonable request.

## Abbreviations

CAPL-2: Canadian Assessment of Physical Literacy second edition
DAPL: the Danish Assessment of Physical Literacy
HRQoL: Health-Related Quality of Life
MVPA: Moderate- to vigorous intensity physical activity
PA: Physical activity
PL: Physical literacy
SDQ: Strengths and Difficulties Questionnaire
SEM: Structural Equation Modelling
SES: Socioeconomic status
