# Supplementary material for: Associations between children’s physical literacy and well-being: is physical activity a mediator?
Source: BMC Public Health. 2022 Jun 29;22:1267. doi: 10.1186/s12889-022-13517-x (PMC9244357; doi:10.1186/s12889-022-13517-x)
Supplement: Supplementary file 1 — Additional file 1. [file 12889_2022_13517_MOESM1_ESM.docx]

STROBE Statement—Checklist of items that should be included in reports of ***cross-sectional studies***

|  | Item No | Recommendation |
| --- | --- | --- |
| **Title and abstract** | 1 | (*a*) Indicate the study’s design with a commonly used term in the title or the abstract |
|  |  | (*b*) Provide in the abstract an informative and balanced summary of what was done and what was found |
| Introduction | | |
| Background/rationale | 2 | Explain the scientific background and rationale for the investigation being reported |
| Objectives | 3 | State specific objectives, including any prespecified hypotheses |
| Methods | | |
| Study design | 4 | Present key elements of study design early in the paper |
| Setting | 5 | Describe the setting, locations, and relevant dates, including periods of recruitment, exposure, follow-up, and data collection |
| Participants | 6 | (*a*) Give the eligibility criteria, and the sources and methods of selection of participants |
| Variables | 7 | Clearly define all outcomes, exposures, predictors, potential confounders, and effect modifiers. Give diagnostic criteria, if applicable |
| Data sources/ measurement | 8* | For each variable of interest, give sources of data and details of methods of assessment (measurement). Describe comparability of assessment methods if there is more than one group |
| Bias | 9 | Describe any efforts to address potential sources of bias |
| Study size | 10 | Explain how the study size was arrived at |
| Quantitative variables | 11 | Explain how quantitative variables were handled in the analyses. If applicable, describe which groupings were chosen and why |
| Statistical methods | 12 | (*a*) Describe all statistical methods, including those used to control for confounding |
|  |  | (*b*) Describe any methods used to examine subgroups and interactions |
|  |  | (*c*) Explain how missing data were addressed |
|  |  | (*d*) If applicable, describe analytical methods taking account of sampling strategy |
|  |  | (*e*) Describe any sensitivity analyses |
| Results | | |
| Participants | 13* | (a) Report numbers of individuals at each stage of study—eg numbers potentially eligible, examined for eligibility, confirmed eligible, included in the study, completing follow-up, and analysed |
|  |  | (b) Give reasons for non-participation at each stage |
|  |  | (c) Consider use of a flow diagram |
| Descriptive data | 14* | (a) Give characteristics of study participants (eg demographic, clinical, social) and information on exposures and potential confounders |
|  |  | (b) Indicate number of participants with missing data for each variable of interest |
| Outcome data | 15* | Report numbers of outcome events or summary measures |
| Main results | 16 | (*a*) Give unadjusted estimates and, if applicable, confounder-adjusted estimates and their precision (eg, 95% confidence interval). Make clear which confounders were adjusted for and why they were included |
|  |  | (*b*) Report category boundaries when continuous variables were categorized |
|  |  | (*c*) If relevant, consider translating estimates of relative risk into absolute risk for a meaningful time period |
| Other analyses | 17 | Report other analyses done—eg analyses of subgroups and interactions, and sensitivity analyses |
| Discussion | | |
| Key results | 18 | Summarise key results with reference to study objectives |
| Limitations | 19 | Discuss limitations of the study, taking into account sources of potential bias or imprecision. Discuss both direction and magnitude of any potential bias |
| Interpretation | 20 | Give a cautious overall interpretation of results considering objectives, limitations, multiplicity of analyses, results from similar studies, and other relevant evidence |
| Generalisability | 21 | Discuss the generalisability (external validity) of the study results |
| Other information | | |
| Funding | 22 | Give the source of funding and the role of the funders for the present study and, if applicable, for the original study on which the present article is based |

*Give information separately for exposed and unexposed groups.

**Note:** An Explanation and Elaboration article discusses each checklist item and gives methodological background and published examples of transparent reporting. The STROBE checklist is best used in conjunction with this article (freely available on the Web sites of PLoS Medicine at http://www.plosmedicine.org/, Annals of Internal Medicine at http://www.annals.org/, and Epidemiology at http://www.epidem.com/). Information on the STROBE Initiative is available at [www.strobe-statement.org](http://www.strobe-statement.org).

**1. Title and abstract**:

*a)* Associations between children’s physical literacy and well-being: is physical activity a mediator?

*b)* See abstract, page 3 (line 29-51) in manuscript

**Introduction**

**2. Background/rationale:** See page 4-5 (line: 53-90) in manuscript.

**3. Objectives.**

Line 80-84 in the manuscript reads:

Therefore, the objectives of this study are to a) investigate the association between PL and different aspects of psychosocial and physical well-being and b) investigate to what extent the associations are mediated by level of MVPA. We hypothesized that children’s PL would be associated with their daily level of PA and well-being and that the relation between PL and well-being would be partly mediated by daily PA (the hypothesized paths are shown in Figure 1).

**Methods**

**4. Study design**

Line 93-97 in the manuscript reads:

This is a cross-sectional study, that uses participants from the DAPL project. The complete design and methodology of the DAPL study are described elsewhere (28), and thus only variables used in the analysis of this paper are described in the following methods section. Briefly, the DAPL study aimed to translate and adapt the Canadian Assessment of Physical Literacy second edition, CAPL-2 (29,30), into Danish language and context and assess the psychometric properties.

**5. Setting:**

Line 97-108 in the manuscript reads:

Data collection was carried out from January to December 2020 at 12 schools in the Eastern part of Denmark (Sealand). Efforts were made to ensure representability by including schools from districts with different distributions of socioeconomic and ethnic backgrounds.

The DAPL measurement protocol was carried out in line with the original CAPL-2 manual (https://www.capl-eclp.ca/). Physical tests and questionnaires were administered during two consecutive physical education (PE) classes, by trained research assistants. In the week in between the test days, pupils wore an accelerometer (for 8 days) to measure MVPA. On the evening of the day before the first PE class and the evening of the last PE class, text messages were sent to the parents with a link to two questionnaires measuring children’s well-being and parental socioeconomic status.

**6. Participants:**

*(a)* Line 111-115 reads:

Nineteen schools were contacted directly or through municipalities of which 14 agreed to participate and two withdrew because of COVID-19. Fifty-two classes from 1^st^ to 6^th^ grade participated in the study. Of the 1144 invited pupils, 948 provided parental consent (mean age 10.2 years) to participate in the study (83% consent rate) and 646 had data on one or more variables in the SEM models and were included in the study.

**7. Variables:**

Line 248-249 and line 255 in the manuscript reads:

Associations between PL, MVPA, physical well-being, and the different aspects of psychosocial well‐being (see hypothesized model in Figure 1).

Adjustments were made for sex, age, and SES.

**8. Data sources/ measurement:**

See line 138-238 in manuscript.

**9. Bias:**

Line 250-251 and line 292-294 in the manuscript reads:

We used maximum likelihood estimation with robust standard errors (MLR) to estimate missing values, which minimises selection bias.

To assess if missing data were associated with a systematic bias, we compared participants with missing data in the well-being measures due to parents failing to fill in the questionnaire (the largest source of dropout) with participants with complete data on well-being (Table 2).

**10. Study size:**

Line 112-115 and line 273 in the manuscript reads:

Fifty-two classes from 1st to 6th grade participated in the study. Of the 1144 invited pupils, 948 provided parental consent (mean age 10.2 years) to participate in the study (83% consent rate) and 647 had data on one of the outcome variables in the SEM models and were included in the study.

The SEM models only include participants with data for the outcome variable.

**11. Quantitative variables:**

Variables used in the analysis were continuous or dichotomous (i.e. sex). No grouping was used. The distributions of study variables were inspected visually and were considered to be normally distributed. The calculation of the raw data into the final variables/measures used in the analysis is explained for each measure in line 142-245.

**12. Statistical methods:**

Line 241-257 in the manuscript reads:

*(a)* Descriptive statistics, unpaired t-tests, bivariate correlations and, robust Cronbach’s alpha estimations were done in SPSS 25.0 (50). McDonald’s Omega (ML) estimations were calculated in SPSS using Andrew Hayes’ macro (51). Reliability was examined for the psychometric subscales, as well as for the three combined scales with values above 0.7 considered acceptable (52,53). In the case where individual scales are used as outcome variables (i.e. physical well-being and pro-social scale), and since these are psychological latent variables measured by only five items, values above 0.6 were considered acceptable (54,55).

Associations between PL, MVPA, physical well-being, and the different aspects of psychosocial well‐being (see hypothesized model in Fig. 1) were investigated through structural equation modelling (SEM) in Mplus (56). We used maximum likelihood estimation with robust standard errors (MLR) to estimate missing values, which minimises selection bias. All models were adjusted for clustering effect by school classes. The distributions of study variables were inspected visually and were normally distributed. We followed recommended criteria for a good model fit: Tucker-Lewis index (TLI > 0.95), comparative fit index (CFI > 0.95), and root mean square error of approximation (RMSEA < 0.06) (Hu & Bentler, 1999; Schermelleh-Engel et al., 2003). Adjustments were made for sex, age, and SES. Covariation between all exogenous variables was allowed. Significance tests were 2-tailed and P-values below 0.05 were considered statistically significant.

*(b)* No sub groups or interactions were investigated

*(c)* We used maximum likelihood estimation with robust standard errors (MLR) to estimate missing values.

*(d)* All models were adjusted for clustering effect by school classes.

*(g)* Sensitivity analysis was carried out. See line 292- 298 in the manuscript.

**Results**

**13. Participants**

*(a)* Line 112-115 in the manuscript reads:

Fifty-two classes from 1^st^ to 6^th^ grade participated in the study. Of the 1144 invited pupils, 948 provided parental consent (mean age 10.2 years) to participate in the study (83% consent rate) and 647 had data on one of the outcome variables in the SEM models and were included in the study.

*(b)* Line 292-293 and line 456-459 in the manuscript reads:

..missing data in the well-being measures due to parents failing to fill in the questionnaire (the largest source of dropout).

Missing data in the PL elements were mostly due to children not attending school on one or both test days. Missing data in the PA measure were due to children removing the monitor after a few days, and to children who lost the monitor or forgot to return it

*(c)* Not used.

**14. Descriptive data**

*(a)* See table 1

*(b)* Missing data (total sample, n = 647):

PL = 160

MVPA = 156

SES = 71

*(c)* not relevant

**15. Outcome data:** Five outcome variables: Physical Well-being, Externalizing score, Internalizing score, Total difficulties score, Prosocial score

**16. Main results:**

*(a)* Please see table 4 and 5 in manuscript. Sex, age and SES were chosen as confounders because they are affecting levels MVPA and PL.

*(b)* Not relevant, as no continuous variables were categorized.

*(c)* Not relevant.

**17. Other analyses:**

No other analysis was done.

**Discussion**

**18. Key results:**

Line 373-377 and line in the manuscript reads:

The results of this study indicate that PL is associated with important aspects of children’s well-being. First, we observed a positive moderate association between PL and physical well-being which was partly mediated by MVPA. The observed association between MVPA and physical well-being was positive and moderate indicating that PL is important for children’s physical well-being and that some of this relationship works through the level of PA.

(Line 384-385) Secondly, we observed a beneficial association between PL and all aspects of psychosocial well-being, with β-values ranging from .21 to -.32, and with no mediating role of MVPA.

(Line 402-406) Together, the non-significant or detrimental associations between MVPA and different aspects of psychosocial well-being, and the beneficial association between PL and psychosocial well-being with or without controlling for MVPA in the model indicate that PL may have a positive impact on children’s general well-being. However, studies with an experimental design are needed to confirm this.

**19. Limitations:**

Line 450-460 in the manuscript reads:

The amount of missing data should be considered a limitation. The highest amount of missing data was in the outcome variables about well-being (68% response rate). This might be due to that these questionnaires were sent to the parents on the last day of the data collection period, however, the response rate for the questionnaire sent on the first day was only a little better (78 %). From the dropout analysis (Table 2) it was evident that the participants with missing data in the outcome variables scored 7.5 % lower in PL but did not differ in MVPA. Missing data in the PL elements were mostly due to children not attending school on one or both test days. Missing data in the PA measure were due to children removing the monitor after a few days, and to children who lost the monitor or forgot to return it. Nevertheless, for such direct measures used in this study the final sample size is rather large and should be considered a strength.

(Line 464-466) However, when using accelerometers alone, information about context, setting, and characteristics of the activities are absent, which could be important to understand the association between PA and psychosocial well-being.

(Line 467-475) The assessment of physical and psychosocial well-being also has some limitations. Physical well-being was assessed by the subdomain of the KIDSCREEN consisting of items that are also part of the construct PA, which perhaps makes it less suitable for exploring associations to MVPA.

The SDQ questionnaire used to measure psychosocial well-being has been criticized for being less suitable to measure the variance within a normal/healthy sample. This instrument was first developed to identify children with difficulties (43), which introduces the risk of a flooring effect, and thus loss of variance, when used in a community sample. Further, the reliability values for the individual prosocial scale were below acceptable values and thus, results related to this outcome should be considered with additional caution.

(Line 481-485) The main limitation of the study is perhaps the cross-sectional design, which introduces uncertainty about the direction or causality of the association. We must consider the possibility that children that thrive in general also thrive in the context of PA’s and thus have higher motivation, confidence, motor skills, etc. Research studies with longitudinal and experimental designs are needed to inform us on the direction of the associations examined in this study.

**20. Interpretation:**

Line 488-524 in the manuscript reads:

This study contributes to the scarce literature on associations between PL and health. The findings bring novel knowledge about the relation between children’s PL and physical and psychosocial well-being and the mediating role of PA. We hypothesized, that PL was associated with physical well-being and aspects of psychosocial well-being in children and found positive beneficial associations for all investigated associations. We further hypothesized, that PA had a mediating role in the associations, but found that this was only the case for the relationship between PL and physical well-being. Results from this study contribute to the existing evidence that PL is related to several health outcomes (17) by indicating that PL is important for children’s psychosocial well-being beyond its association to MVPA.

However, studies with an experimental design are needed to confirm this.

**21. Generalisability:**

Line 441-447 in the manuscript reads:

First, findings from this study respond to the call for more research on the link between PL and physical, psychological, and social health, and provide more support for the assumption that PL is a determinant of health. Second, the null finding of MVPA on aspects of psychosocial well-being along with the positive relation to PL supports the idea that helping children develop their PL is more beneficial for general long-term health than a narrow focus on increasing their levels of PA. This speaks to a need of shifting the focus from the amount and intensity of PA to quality and enjoyment when encouraging children to move.

**Other information**

**22. Funding:**

Line 533-535 in the manuscript reads:

This study was supported by the TrygFonden (ID: 125640) and The Industrial Researcher Program, Innovation Fund Denmark (9065-00060B). The funders have not been involved in the study design, analyses, interpretation, writing, or decision to submit this paper.
